# Role of FMS-Like Tyrosine Kinase 3 (FLT3) Inhibitors in a Patient With T/Myeloid Acute Leukemia With an FLT3 Mutation

**DOI:** 10.7759/cureus.105695

**Published:** 2026-03-23

**Authors:** Nicholas Elias, Ahsun Siddiqui, Talal Bazzi, Daniel Lebovic

**Affiliations:** 1 Internal Medicine, Henry Ford St. John Hospital, Detroit, USA; 2 Hematology/Oncology, Henry Ford St. John Hospital, Detroit, USA

**Keywords:** flt3-itd mutation, midostaurin, mixed-phenotype acute leukemia (mpal), t/myeloid leukemia, tyrosine kinase inhibitor

## Abstract

Mixed-phenotype acute leukemia (MPAL) is a rare and aggressive hematologic malignancy characterized by the co-expression of myeloid and lymphoid markers, posing diagnostic and therapeutic challenges. The presence of FMS-like tyrosine kinase 3 internal tandem duplication (FLT3-ITD) mutation, common in acute myeloid leukemia (AML) but rare in MPAL, introduces further uncertainty regarding optimal treatment strategies, particularly regarding the use and timing of FLT3 inhibitors. We report a case of a 59-year-old man who presented with marked leukocytosis and systemic symptoms. Diagnostic workup, including flow cytometry and bone marrow biopsy, confirmed MPAL, T-cell/myeloid type, with an FLT3-ITD mutation. The patient was initially treated with a hybrid induction regimen of FLAG-IDA (fludarabine, arabinofuranosyl cytidine, granulocyte colony-stimulating factor (G-CSF)-idarubicin) plus vincristine and prednisone, followed by reinduction with decitabine and venetoclax due to persistent disease. Morphologic and immunophenotypic remission was achieved, and the patient was bridged to allogeneic stem cell transplantation. Midostaurin, an FLT3 inhibitor, was introduced in the post-remission and pre-transplant consolidation phases to reduce toxicity. This case highlights the diagnostic complexity and therapeutic uncertainty associated with FLT3-mutated MPAL. While FLT3 inhibitors are standard in AML, their role in MPAL remains undefined, with few case reports being published on this topic. Our case highlights the importance of individualized treatment planning and supports further research into the optimal timing and integration of targeted therapy in MPAL. FLT3-ITD mutation in MPAL presents a unique therapeutic dilemma. In this case, the delayed introduction of midostaurin was favored to minimize toxicity during induction. Ongoing studies are needed to determine the best treatment strategies and timing for targeted therapies in this rare leukemia subtype.

## Introduction

Acute leukemia is a form of cancer that affects the blood and the bone marrow, leading to an unregulated production of abnormal white cells. This hematological malignancy most commonly arises from either myeloid or lymphoid progenitor cells; however, in certain cases, leukemic blasts display immunophenotypic and/or genetic features characteristic of both acute lymphoblastic leukemia (ALL) and acute myeloid leukemia (AML) [1,2]. These acute leukemias are now regarded as mixed-phenotype acute leukemias (MPAL), which are associated with poor outcomes due to a lack of studies on a standardized treatment regimen and controversy over whether AML-based, ALL-based, or combined AML/ALL-based approaches will provide the best outcome [1,3]. FMS-like tyrosine kinase 3 (FLT3) gene mutations occur in roughly 30% of all AML cases, with the internal tandem duplication (FLT3-ITD) representing about 25% of all AML cases. FLT3-ITD is a common driver mutation and is associated with a poor prognosis in patients with AML, due to its effect of enhancing proliferation and reducing apoptosis [4]. There are several tyrosine kinase inhibitors that are approved for the treatment of FLT3-mutated AML, including midostaurin and gilteritinib (type I inhibitors) and quizartinib (type II inhibitors), along with induction chemotherapy. However, in patients with MPAL and FLT3 mutation, data are lacking on whether an FLT3 inhibitor should be used and when, in the treatment process, clinicians should use it, as its presence provides an additional therapeutic target that could potentially be exploited. In this case presentation, we discuss a 59-year-old man with FLT3-ITD-mutated T/myeloid acute leukemia and how we treated him. 

## Case presentation

A 59-year-old man initially presented to the hospital after he was found to have an elevated white blood cell count when seen by his primary care provider (PCP). At the time of presentation, the patient reported a 10 lb weight loss in the past two weeks, shortness of breath, and night sweats. Physical exam at the time of admission revealed no significant findings. Initial labs at the time of the patient's presentation are shown in Table 1. These labs are concerning for active malignant proliferation, abnormal leukemic cells, and increased cell turnover.

**Table 1:** Initial presenting lab results WBC: white blood cell; RBC: red blood cell; MCV: mean corpuscular volume; MCH: mean corpuscular hemoglobin; MCHC: mean corpuscular hemoglobin concentration; RDW: red blood cell distribution width; PMN: polymorphonuclear leukocyte

Lab	Result	Reference range
WBC	60.55 K/mcL	4-11 K/mcL
RBC	2.64 million/mcL	4.2-5.9 million/mcL
Hemoglobin	9.3 g/dL	13.5-17.5 g/dL
Hematocrit	27%	41-53%
MCV	102.3 fL	80-100 fL
MCH	35.2 pg	27-33 pg
MCHC	34.4 g/dL	32-36 g/dL
RDW	15.3%	11.5-14.5%
Neutrophils (auto %)	1%	40-70%
Lymphocytes (auto %)	41%	20-40%
Eosinophils (auto %)	1%	0-5%
Absolute PMN	0.61 K/mcL	1.5-8 K/mcL
Absolute lymphocytes	58.13 K/mcL	1-4 K/mcL
Absolute eosinophils	0.61 K/mcL	0-0.5 K/mcL
Atypical lymphocytes	55%	0%

A computed tomography (CT) of the abdomen/chest/pelvis with IV contrast done on admission revealed a small fatty umbilical hernia, cholelithiasis, and no evidence of splenomegaly or adenopathy. Flow cytometry was subsequently performed, detecting approximately 92% blasts with dual expression of T-lineage and myeloid markers; the immunophenotyping results are shown in Table 2 and are consistent with T/myeloid MPAL.

**Table 2:** Results of the flow cytometry immunophenotyping of leukemic cells

Markers	Result
Tdt, CD34, cytoplasmic CD3, CD117, CD2, CD7, CD13	Positive
CD33	Partially positive
Surface CD3, CD4, CD5, CD8, CD1a, CD19, CD20, Surface light chains, CD14, CD64, CD11b, CD11c	Negative

**Figure 1:**
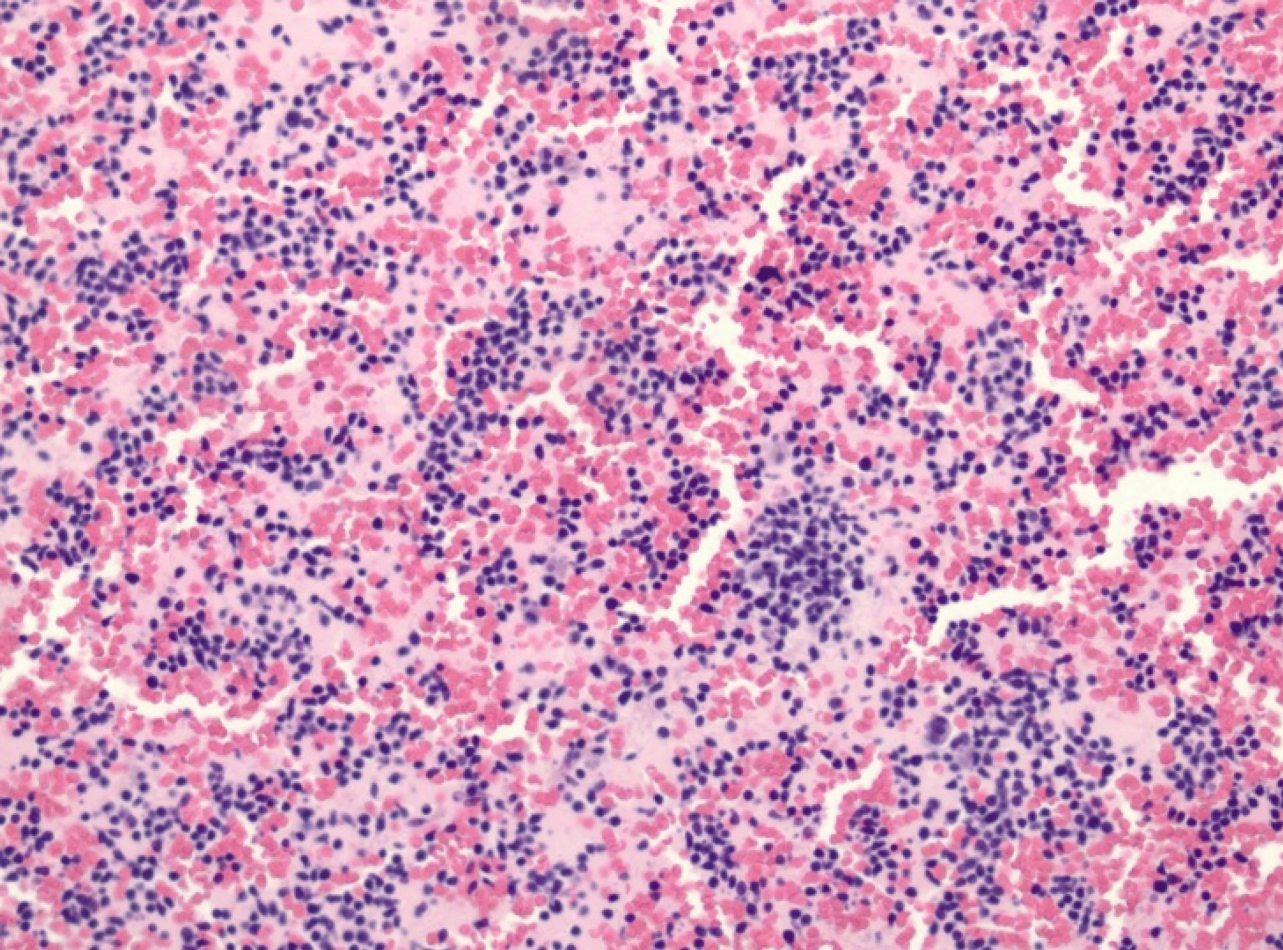
Hypercellular bone marrow with 95% cellularity

**Figure 2:**
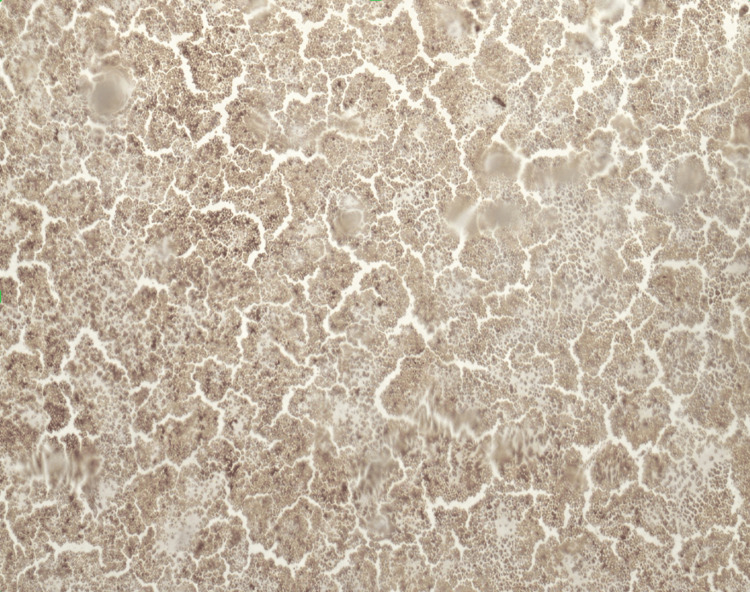
CD34 staining, which highlights blasts in approximately 95% of the marrow

A bone marrow biopsy (Figures 1-2) was then performed with CD34 staining, showing MPAL with 95% blasts, with immunophenotyping results in Table 3, supporting persistent T-lineage differentiation consistent with T/myeloid MPAL. Blasts are positive for CD34, CD3, CD200, HLA-DR, and partial expression of CD2 and CD7. Flow cytometry showed 92% blasts with dual T-lineage and myeloid marker expression. The patient was also found to have an FLT3-ITD mutation, which is very unusual for MPAL. After discussion with multiple tertiary referral and transplant centers, we decided to start midostaurin as part of his consolidation and post-transplantation regimen to decrease grade III and IV toxicities. The patient was then started on FLAG-IDA (fludarabine, arabinofuranosyl cytidine, granulocyte colony-stimulating factor (G-CSF)-idarubicin) plus vincristine (VCR) and prednisone receiving fludarabine 30 mg/m² on days 1-4, cytarabine 2000 mg/m² on days 1-4, idarubicin 12 mg/m² on days 2-4, vincristine 1.4 mg/m² on days 1, 8, 15, and 22, and prednisone 60 mg/m² on days 1-21.

**Table 3:** Bone marrow immunophenotyping results

Markers	Result
CD34, CD3, CD200, HLA-DR	Positive
CD2, CD7	Partially positive

**Figure 3:**
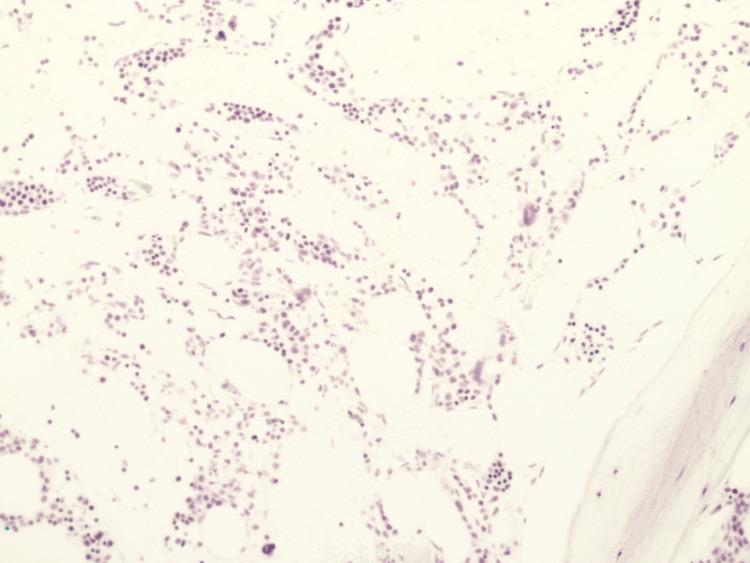
Cellularity of the bone marrow, which is approximately 40%

**Figure 4:**
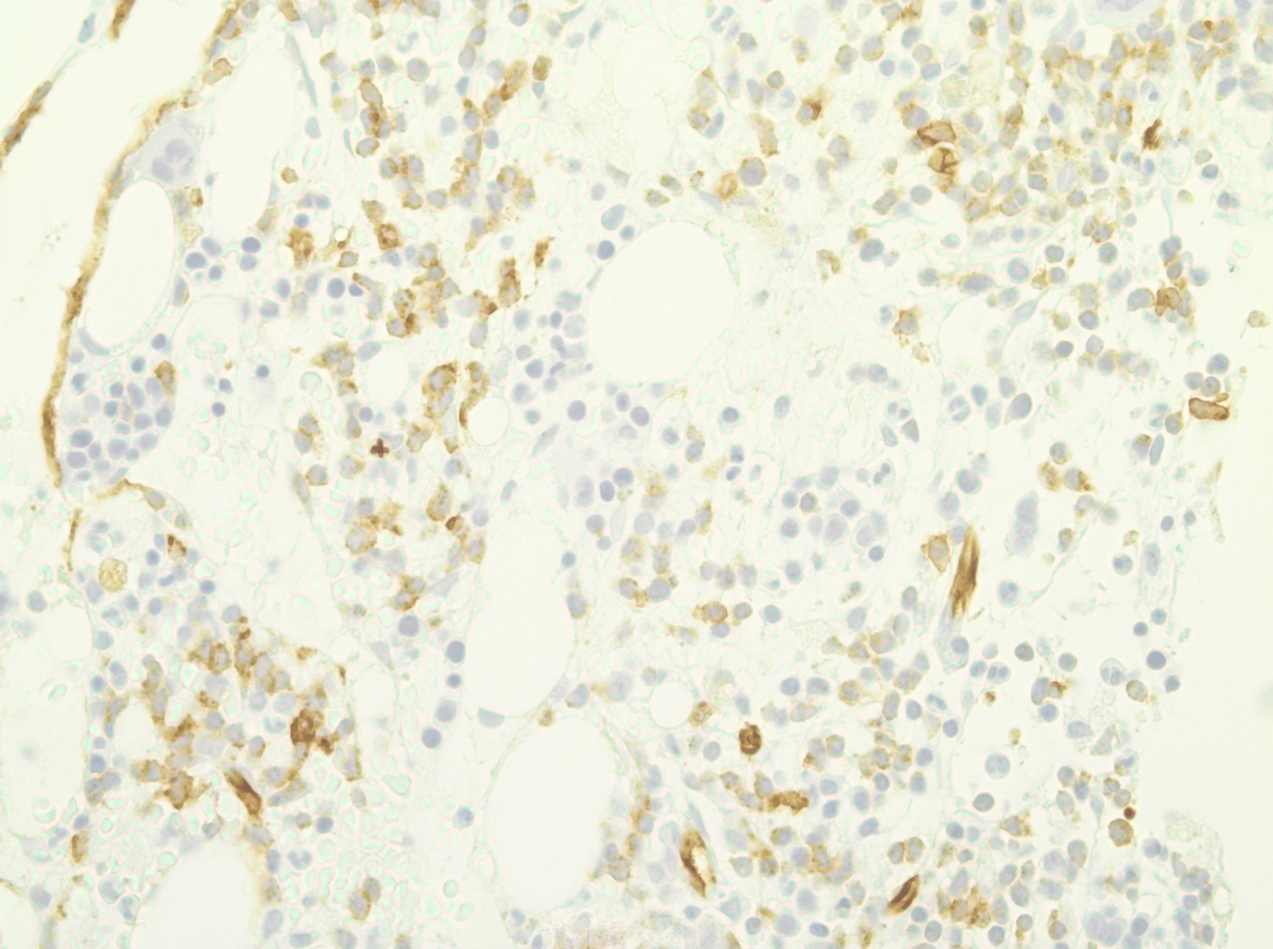
CD34 staining, which highlights blast cells accounting for 20%

Following the completion of his initial chemotherapy regimen and reaching nadir, a repeat bone marrow biopsy showed cellularity of around 40% and persistent leukemia with about 20 blasts (Figures 3-4).

**Figure 5:**
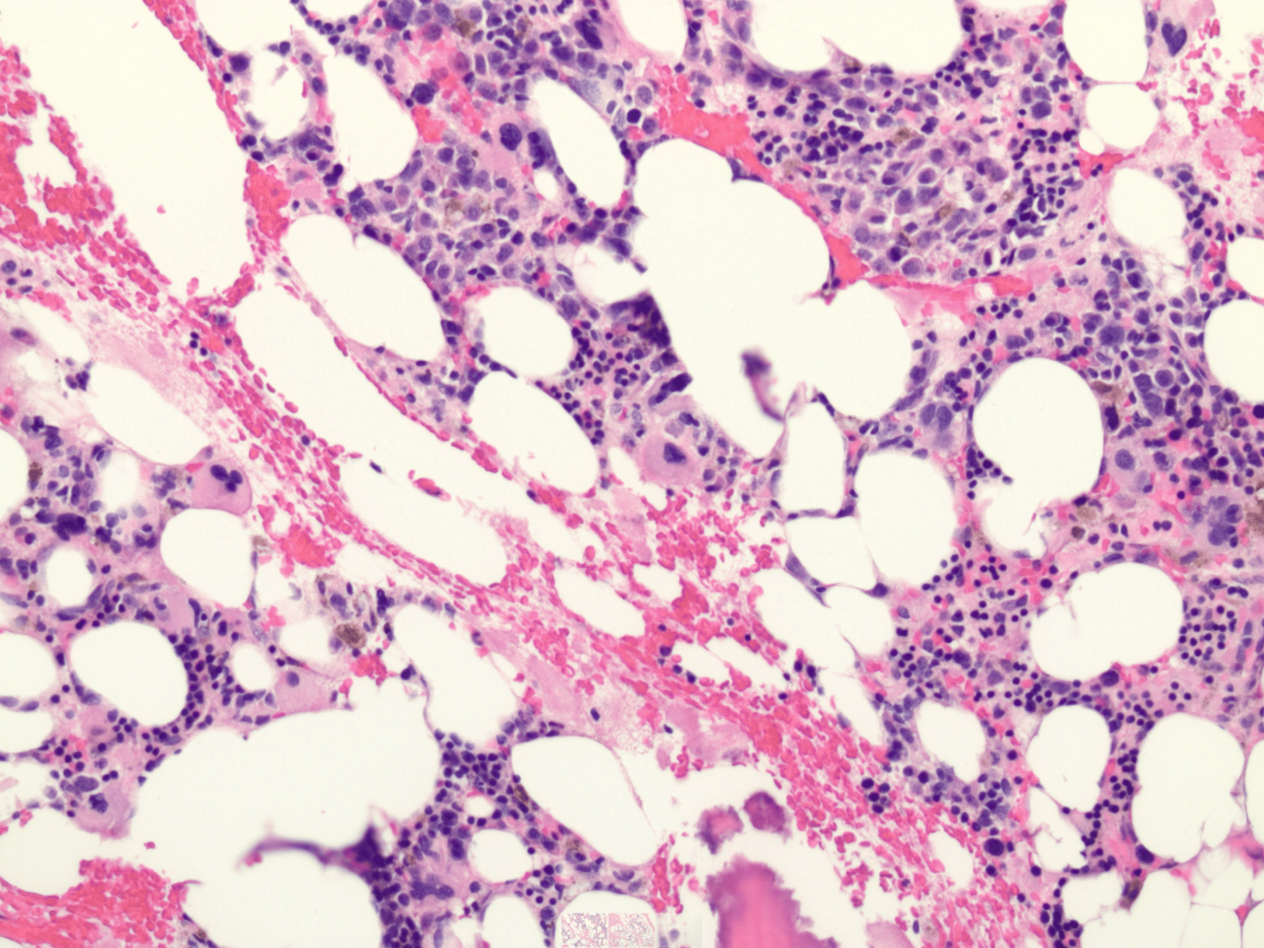
Cellularity of 30% with no evidence of acute myeloid leukemia

**Figure 6:**
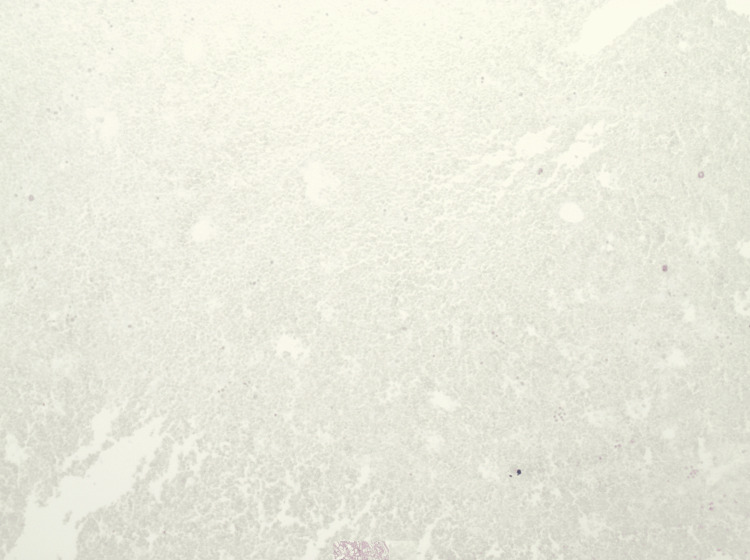
CD34 stains, which are negative, and no blasts are seen

The patient then underwent reinduction with decitabine 40 mg on days 1-10 and venetoclax 400 mg on days 7-10. A repeat bone marrow biopsy after reinduction demonstrated no morphological or immunophenotypic evidence of acute leukemia (Figures 5-6).

The patient underwent a second cycle of decitabine and venetoclax as a bridging treatment while he waited for allogeneic stem cell transplantation. He went on to receive his allogeneic peripheral blood stem cell transplantation and is currently doing well on gilteritinib for maintenance therapy, living disease-free.

## Discussion

MPAL represent roughly 1-3% of all acute adult leukemias [3]. An understanding of the mutations of MPAL poses significant diagnostic and therapeutic challenges due to its ambiguous immunophenotypic profile. Distinguishing MPAL from acute leukemias with aberrant antigen expression or lineage infidelity requires comprehensive immunophenotyping by flow cytometry and corroborative cytogenetic or molecular studies [4]. However, the identification of key mutations such as FLT3 can offer new avenues for individualized therapy. 

Identification of the FLT3 mutation modifies the way patients with AML are treated and could play a role in MPAL as the mutation operates similarly in both. FLT3 plays a significant role, leading to cell proliferation and anti-apoptosis, which results in a poor prognosis for those patients. However, as discussed earlier, targeted therapy for this mutation has been established [5]. With the initiation of midostaurin along with chemotherapy in those who were positive for the FLT3 gene mutation, a 22% lower risk of death was seen [6]. With this medication being so promising, it begs the question of whether or not this tyrosine kinase inhibitor should be used in patients with MPAL who are positive for this gene, and when to start it.

Chemotherapy regimens used to treat acute leukemias are associated with side effects, particularly marked myelosuppression. Patients can also commonly experience a range of debilitating side effects, including fatigue, nausea, vomiting, mucositis, alopecia, and increased susceptibility to infections. This presents a significant difficulty in many chemotherapy regimens as they may become less tolerable to patients. Many chemotherapy medications have similar side effects, and in this case, we believe that introducing the FLT3-ITD inhibitor midostaurin early on in treatment would have introduced many overlapping toxicities. However, it is unknown whether the addition of this medication would have been tolerable by the patient during induction therapy. With the addition of midostaurin in both the induction phase and the consolidative phase, success in treatment was seen without significant adverse events in patients with FLT3-positive mutations [2]. This case focuses on whether introducing midostaurin earlier in treatment would allow for increased rates of treatment success.

T/myeloid acute leukemia is an aggressive and difficult form of AML to treat. Research into the appropriate treatment regimen is limited. Advances in immunophenotyping, molecular diagnostics, and targeted therapy are gradually transforming the clinical approach to MPAL, but further prospective studies are essential to further establish standardized treatment protocols and improve patient outcomes. As the understanding of treatment for MPAL broadens, studies should focus on the appropriate timing of when to introduce targeted therapy for patients who are also harboring a targetable genetic mutation/alteration. 

## Conclusions

FLT3 inhibitors work by targeting and blocking the action of FLT3, which promotes the proliferation of leukemic cells, downregulating leukemic cell proliferation. The use of midostaurin alongside chemotherapy carries risks, particularly when considering the potential for cumulative toxicity. In our case, the medication was held and recommended to be used later on in this patient's course to allow us to decrease the risk of grade III and IV toxicities. However, as progress is made in treating MPAL, the question remains when introducing targetable therapy will be most beneficial in patients' relapse-free and overall survival. 
